# Laryngeal tuberculosis: about 04 cases

**DOI:** 10.11604/pamj.2023.45.193.5325

**Published:** 2023-08-31

**Authors:** Abdelilah Mouhsine, Ahmed Belkouch, Hind Temsamani, El Mehdi Atmane, Redouane Rokhssi, Youssef Berrada, Saad Zidouh, Lahcen Belyamani, Fouad Benariba, M'barek Mahfoud, Abdelghani El Fikri

**Affiliations:** 1 Department of Radiology, Avicenna, Military Hospital, Marrakech, Morocco,; 2 Emergency department, Mohamed V Military Hospital of Instruction, Rabat, Morocco,; 3 Department of ENT and Cervico-Facial Surgery, Mohamed V Military Hospital of Instruction, Rabat, Morocco

**Keywords:** Tuberculosis, extra nodal, ear, nose and throat, larynx, anti-TB drugs

## Abstract

The purpose of this study is to present epidemiological, clinical, radiological, histological characteristics and treatment of laryngeal tuberculosis. It is also aimed at making the point about diagnosis difficulties. This retrospective study was conducted over three years; it concerned 4 cases, 3 males and one female. The average age was 35 years. Three of the 4 cases have had a cervical CT scan. All patients have had a laryngoscopy with biopsy and anatomopathological study. The onset modes of the disease have been progressive for all the patients. Topographical study has shown two epiglottic locations, one at the vocal cords and the other one at the posterior commissure. The diagnosis was orientated in the 3 cases by the CT scan and confirmed by anatomopathological exam in all cases. All patients have received anti-TB drugs with good evolution. The laryngeal location of tuberculosis is unusual. The clinical picture is nonspecific, raising the issue of differential diagnosis with tumor pathology. Sectional imaging and CT scan can guide the diagnosis and a positive diagnosis is often discovered on the occasion of a tumor biopsy of a pseudo-tumor lesion. Treatment is based on anti-TB drugs.

## Introduction

Pulmonary tuberculosis is the most frequent location of tuberculosis. It raises issue of public health and represents the dissemination form of this disease. However, extra pulmonary locations interest now the authors because of its progressive increasing incidence. Ear Nose and Throat (ENT) location is dominated by nodal involvement. Nevertheless, extra nodal localization is not exceptional and clinical picture depends on the affected organ rather than on TB disease. Among extra nodal ENT localizations, laryngeal TB is characterized by clinical polymorphism and often, by misleading aspects raising issue of differential diagnosis with tumor pathology. Therefore, it is rarely mentioned at first and its diagnosis is often a histological surprise. The purpose of this work is to highlight epidemiological, clinical, radiological aspects, diagnosis difficulties, therapeutic modalities from four observations collected in our service.

## Methods

**Sample study:** the study has concerned 4 patients having primitive laryngeal tuberculosis confirmed by histology and collected at medical imaging service of Avicenna Military Hospital of Marrakech.

**Study procedures:** for every file, we raised the following informations: epidemiology: frequency, age, vaccine status, personnel and or/family history of tuberculosis; clinical presentation: ENT symptomatology, existence of fever or other tuberculosis location; investigations: (1) endoscopic and radiological examinations; (2) bacteriological assessment; (3) histological study.

## Results

### Epidemiology

**Frequency:** over a period of 3 years, we collected 15 cases of extra nodal ENT tuberculosis whose 4 cases are laryngeal. Distribution by sex: 03 males and one female, sex ratio was 3. Distribution by age: age of patients varied between 18 and 70 years with an average of 35 years. Tuberculosis history: 2 of our patients (50%) had tuberculosis history, especially pulmonary localization. Vaccine status:3 patients therefore 75% are vaccinated against tuberculosis. Second localization has been found in only one case: 25%, which was concomitant pulmonary tuberculosis with laryngeal involvement.

### Clinical aspects

The onset of symptoms has been progressive in most patients who were all immunologically competent. The clinical picture was dominated by dysphonia, found in all cases. It was permanent, installed progressively and associated with weight loss in 75% of cases. Dyspnea has been found in 50% of cases whereas dysphagia has been observed in only one case (Table 1).

**Table 1:** onset of symptoms

	Numbers of cases	Percentage
**Dysphonia**	4	100
**Dyspnea**	2	50
**Dysphagia**	1	25
**weight loss**	3	75

### Para clinical aspects

Para clinical assessment was based on 2 orientation examinations, endoscopic and radiological ones as well as bacteriological and histological study.

### Diagnostic orientation examination

**Figure 1:**
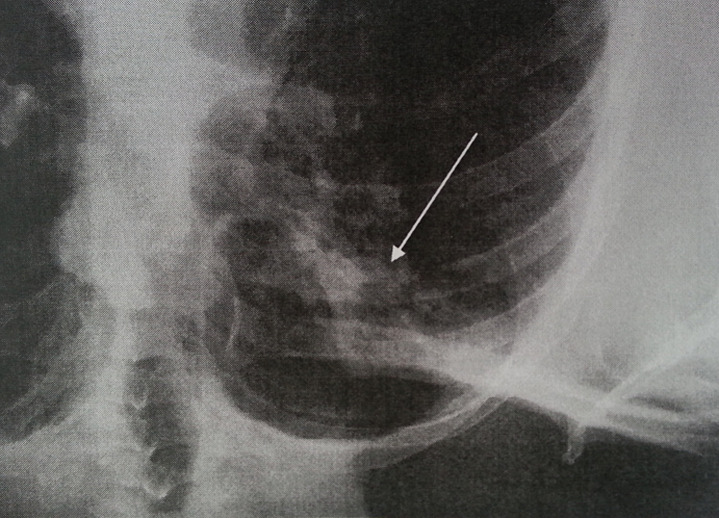
chest radiograph showing reticulo nodular infiltrate of the right apex

**Figure 2:**
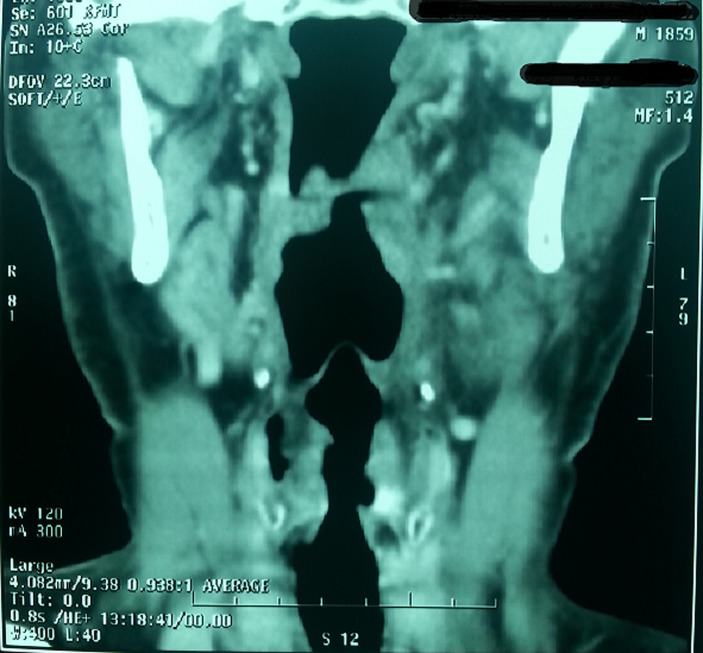
front reconstruction image of laryngeal CT scan objectifying the presence of a thickening, heterogeneous tissue of the left supra glottic laryngeal stage reaching lateral glosso epiglottic ligament and ipsilateral vallicula sidewall

**Figure 3:**
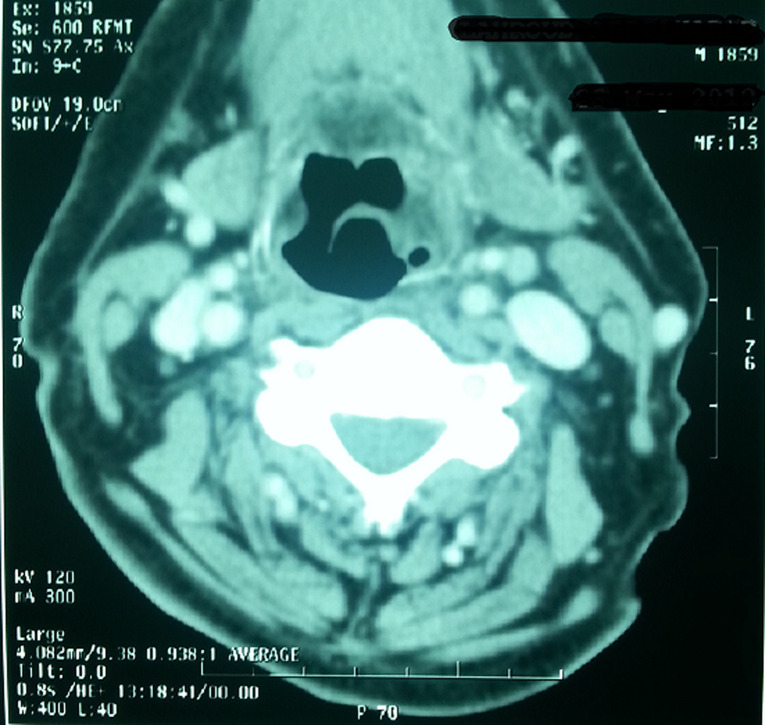
laryngeal CT axial section after injection of contrast material objectifying the presence of a thickening, heterogeneous tissue of the left supra glottic laryngeal stage reaching lateral glosso epiglottic ligament and ipsilateral vallicula sidewall

**Figure 4:**
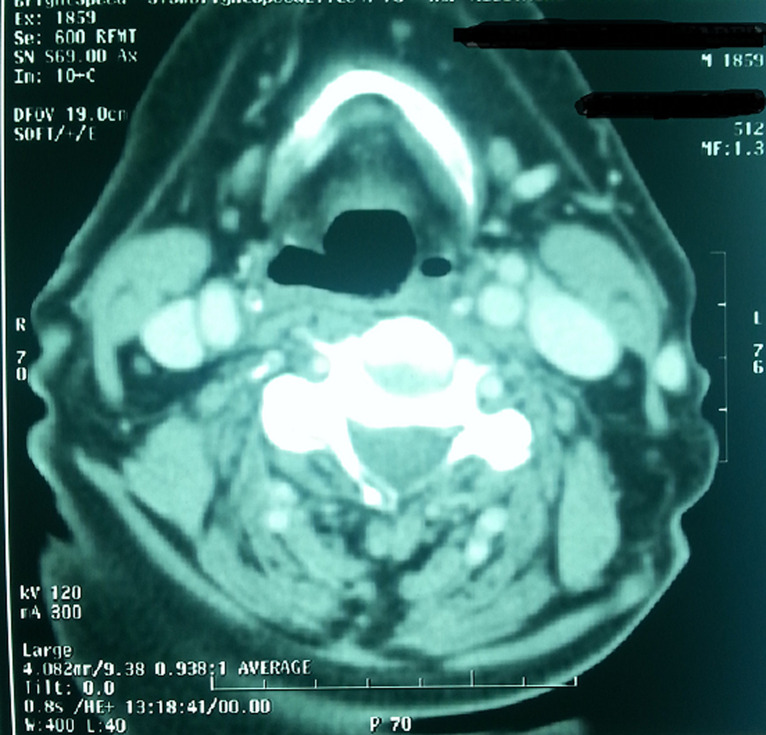
laryngeal CT axial section after injection of contrast objectifying a filling of the left ventricle with collapse of the ipsilateral piriform sinus

**Figure 5:**
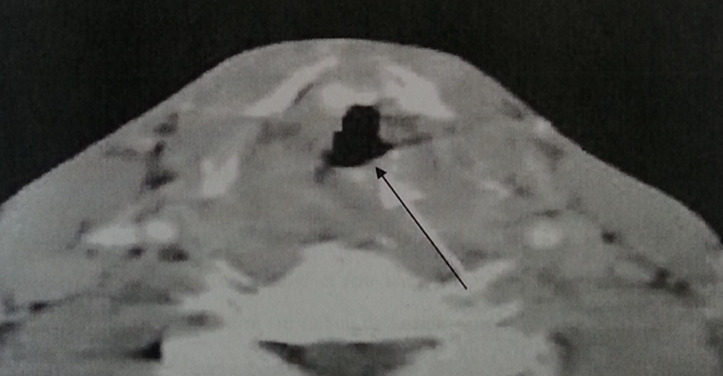
pseudo tumor form of laryngeal tuberculosis

**Figure 6:**
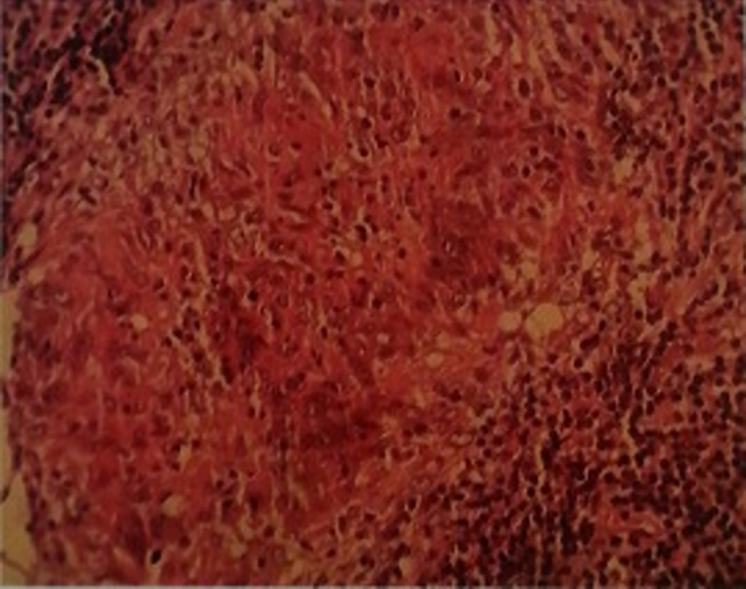
histological sample showing a nasopharyngeal mucosa subject to a granuloma giant with epithelioid cells caseating confirming active tuberculosis

Standard assessment: intradermal reaction to tuberculin (IDR): was positive in 3 cases and negative in one case; C Reactive Protein (CRP): was accelerated in 3 cases; hyperleucocytosis was found in all cases; HIV serology was made in only one case, it was found negative; chest radiograph showed images suggesting pulmonary tuberculosis in one case (Figure 1), whereas in the other patients, it was normal. Radiological examinations: CT scan was performed in 3 cases and showed pseudo tumor aspect of larynx tuberculosis in 3 cases (Figure 2, Figure 3, Figure 4, Figure 5). Direct laryngoscopy showed pseudo tumor lesions in 3 cases and an irregular ulceration aspect in one case, presented with decreasing frequency order as shown in Table 2. In all these cases, larynx cancer has been evoked first of all, except in one case, it was associated with progressing pulmonary tuberculosis. Positive diagnosis has been based on histological confirmation by setting evidence of epithelioid and giant granuloma cell and or bacteriological by setting evidence of acido-alcohol resistant bacilli (AARB) through direct examination or after culture in Lowenstein medium. Diagnosis was based on histological confirmation by highlighting epithelioid and giant cell lesions with caseous necrosis in all cases (Figure 6). The treatment was based on anti-TB drugs, during 6 months. The evolution under treatment was satisfactory. Control after 3 months after the end of treatment showed regression of clinical, endoscopic and radiological signs. Biopsies showed a sterilization of lesions without histological evidence of malignancy. Patients were followed on regular basis without any local recurrence, with a mean follow of 18 months.

**Table 2:** direct laryngoscopy showed pseudo-tumor lesions in 3 cases and an irregular ulceration aspect in one case

localization	Number of cases	Percentage
**epiglottis**	2	50
**Vocal cords**	1	25
**Posterior commeasure**	1	25

## Discussion

Extra pulmonary tuberculosis localization represents currently 20 to 40% of all tuberculosis involvements [1]. Extra nodal ENT tuberculosis remains rare and represents 1.8 % of all tuberculosis localizations [2]. However, this pathology remains underestimated especially in endemic countries such as Morocco. This is because there is no systematic examination of the upper aero digestive tract in case of any tuberculosis involvement. The ENT tuberculosis localization is usually associated with pulmonary forms. The primitive form, although rare is not exceptional [3]. Laryngeal localization is the most frequent, but the pharynx, ear, salivary glands, nose, and thyroid gland, can also be affected with varying degrees. Laryngeal localizations of tuberculosis are rare and represent less than 1% of all manifestations of that disease. Nevertheless, in the upper digestive and airways, the larynx is the most affected site by tuberculosis, reaching a rate of 46% [4,5]. It is rarely isolated, and often is associated with pulmonary tuberculosis which was formerly, the fatal complication leading to death. The age of tuberculosis laryngitis onset was considerably higher, parallel to the level of the population vaccination. Actually, the average age is 50 years, whereas it was 35 in our series.

According to all authors, men are more likely to suffer from this pathology, as it was in our series, which seems to be facilitated by excessive alcohol and tobacco intoxication. Gallas believes that the frequency of tobacco intoxication among patients having laryngeal tuberculosis is 72.6% [6] In our series, we found three smokers (75%), among them 02 were Alcoholics. A history of pulmonary tuberculosis was found in 20.6% of cases in the study of 223 cases of laryngeal tuberculosis made by Gallas. In our series, the history of pulmonary is present in 02 cases (50%). Some authors have reported cases of laryngeal tuberculosis in patients undergoing renal transplantation [7,8]. Laryngeal tuberculosis, unlike pharynx TB, is almost always secondary to pulmonary disease whose lesions are not necessarily in the foreground. The secondary laryngeal involvement can be done either by hematogenous, lymphatic, and /or airway dissemination. Isolated localization is very rare. It could be for some persons, reactivation of a laryngeal outbreak during the dissemination phase of an ancient primary infection. For others, it could be an exogenous infection closer to a primary tuberculosis infection [9-11].

The described classic symptomatology of laryngeal tuberculosis has changed. Currently, laryngeal tuberculosis no longer occurs at the final stage of the disease. Most often, it reveals it and lung injury comes after [5]. The diagnosis period is generally slow, variable from 1 to 12 months, with an average of three months. The symptoms of laryngeal tuberculosis are usually dysphonia (76 to 96% of cases) [12]. It is a progressive and banal dysphonia, present at varying degrees; sometimes tense, leading to a dark voice. It can be related to laryngeal involvement or recurrent paralysis from mediastinal origin in the case of combined pulmonary tuberculosis. Coughing is variable, depending on lesions. It can be a dry cough of laryngeal origin or it can be a productive cough with mucopurulent secretions, sometimes hemoptoic-related lung injury. According to Gallas, it is indicative of the disease in 40.5% of cases [6,11]. Dysphagia is common (40%) and typical [12]. It can be linked to the extension of the laryngeal curb lesions. For Gallas [6], it is 36.8%. In our series, it is found in 2 cases, one patient had dysphagia (25%). Dyspnea is found in less than 20% of cases in literature and rarely requires a tracheotomy [12,13]. In our series, it was found in 2 cases (50%). In children, stridor may be the only clinical sign in case of laryngeal tuberculosis [11]. Poor general condition and weight loss are found in nearly half cases for Gallas.

Laryngoscopy is used to specify the exact topography and to show the lesion's aspects and carry out biopsies for a histological study and bacteriological examination if TB is suspected. Today, laryngeal tuberculosis causes diffuse affection of anatomical elements of the larynx. It predominantly affects vocal cords, followed by the laryngeal vestibule and the subglottic region [14]. Laryngeal tuberculosis can reach one, two or three levels. We can distinguish: the shape of congestive edema, pseudotumor, ulcer budding, papillomatous, infiltrative, and distributive forms. In our series, the tumor-like form is present in 03 cases, 75% and irregular ulcerations in one case, 25%. The definitive diagnosis is based on histological examination of biopsy by highlighting epithelioid giant cell granuloma or bacteriological one, by highlighting AARB. The imagery is based primarily on a computed tomography scan (CT scan), which is useful in the topographic assessment of extra laryngeal extension to clarify the diagnosis of chondritis or the balance of subglottic stenosis at scar stage [11,15]. This examination is rarely asked in the endo laryngeal forms because there is no suggestive radiographic appearance of tuberculosis in this form.

Studies conducted to evaluate CT scan data on laryngeal tuberculosis and to differentiate them from those of carcinoma revealed the following evocative aspects: [16,17] bilateral and diffuse location; thickening of the free edge of the epiglottis; preservation of adipose pre glottal and pre laryngeal space without destruction of the laryngeal architecture. In the case of carcinoma, location often is unilateral with infiltration of preepiglottic and peri laryngeal adipose tissue as well as cartilage destruction and extra laryngeal invasion. Chest radiography should be systematically applied to all laryngeal lesions because lung involvement can be latent. The differential diagnosis is a problem with other affections [1,6,13], mainly with cancer and ordinary laryngitis [6,11,13], TB chemotherapy is currently the preferred treatment for tuberculosis in ENT. The treatment and surveillance (monitoring) should be carried out in collaboration with physiologists. The role of surgical treatment is currently restricted. It is limited to tracheotomy in case of obstructive lesions and ones leading to dyspnea, which is unusual these days. Surgical treatment is also limited to the treatment of squeals such as cicatricial subglottic stenosis [11,15]. The response to treatment is usually favorable with the disappearance of symptoms and inflammatory and exudative lesions before the end of the first month, however, the lack of improvement or relapse raises the question of the possible occurrence of cancer and imposes an endoscopic and histological control [9].

## Conclusion

The laryngeal location of tuberculosis is unusual. The clinical picture is nonspecific, raising the issue of differential diagnosis with tumor pathology. Sectional imaging, CT scan can guide the diagnosis, and a positive diagnosis is often discovered on the occasion of a tumor biopsy of a pseudo tumor lesion. Treatment is based on anti-TB drugs.

### What is known about this topic

- *Tuberculosis most frequent location is the lung;*
- *Tuberculosis becomes a public health issue;*
- *Ear Nose and Throat (ENT) location is dominated by nodal involvement*.

### What this study adds

- *Extra pulmonary locations are unusual;*
- *The clinical picture is nonspecific;*
- *Positive diagnosis is often discovered on the occasion of a laryngeal tumor like biopsy*.
